# A Web-Based Intervention for Social Media Addiction Disorder Management in Higher Education: Quantitative Survey Study

**DOI:** 10.2196/14834

**Published:** 2019-10-02

**Authors:** Huseyin Dogan, Helmi Norman, Amen Alrobai, Nan Jiang, Norazah Nordin, Anita Adnan

**Affiliations:** 1 Bournemouth University Bournemouth United Kingdom; 2 Faculty of Education Universiti Kebangsaan Malaysia Bangi Malaysia; 3 King Abdulaziz University Jeddah Saudi Arabia; 4 Doctorate Support Group Kuala Lumpur Malaysia

**Keywords:** Facebook addiction, intervention features, postgraduate education, social media addiction, obsessive-compulsive disorder (OCD), PLS-SEM analysis

## Abstract

**Background:**

Social media addiction disorder has recently become a major concern and has been reported to have negative impacts on postgraduate studies, particularly addiction to Facebook. Although previous studies have investigated the effects of Facebook addiction disorder in learning settings, there still has been a lack of studies investigating the relationship between online intervention features for Facebook addiction focusing on postgraduate studies.

**Objective:**

In an attempt to understand this relationship, this study aimed to carry out an investigation on online intervention features for effective management of Facebook addiction in higher education.

**Methods:**

This study was conducted quantitatively using surveys and partial least square-structural equational modeling. The study involved 200 postgraduates in a Facebook support group for postgraduates. The Bergen Facebook Addiction test was used to assess postgraduates’ Facebook addiction level, whereas online intervention features were used to assess postgraduates’ perceptions of online intervention features for Facebook addiction, which are as follows: (1) self-monitoring features, (2) manual control features, (3) notification features, (4) automatic control features, and (5) reward features.

**Results:**

The study discovered six Facebook addiction factors (relapse, conflict, salience, tolerance, withdrawal, and mood modification) and five intervention features (notification, auto-control, reward, manual control, and self-monitoring) that could be used in the management of Facebook addiction in postgraduate education. The study also revealed that relapse is the most important factor and mood modification is the least important factor. Furthermore, findings indicated that notification was the most important intervention feature, whereas self-monitoring was the least important feature.

**Conclusions:**

The study’s findings (addiction factors and intervention features) could assist future developers and educators in the development of online intervention tools for Facebook addiction management in postgraduate education.

## Introduction

### Background

Addiction is usually associated with addictive behavior and compulsive engagements of stimuli, such as a psychoactive chemical (eg, alcohol and drugs), despite harmful consequences. Nevertheless, behavioral addiction related to nonconsumption of substances, such as digital addiction, has recently become a topic of much interest. To date, the only psychiatric disorders that have been formally recognized are pathological gambling and internet gaming disorder [1,2]. As such, there is an urgent need for further research in terms of behavioral addiction [3]. As social media has become an essential platform for online communication, several studies have investigated its behavioral effects on excessive usage. Although some researchers have addressed general digital and internet addiction [4] and its psychological effects on loneliness, anxiety, and depression [5], other researchers have focused on addiction of social networking sites (SNSs) such as Facebook [2,5].

As of June 2018, current statistics have indicated that Facebook is the most popular social network worldwide, with over 2 billion monthly active users. Despite its benefits for the Web-based social communication and content consumption, some users develop an excessive usage of Facebook, causing potential negative effects, termed as Facebook addiction disorder [2,5]. Facebook addiction is defined as an addictive behavior caused by an uncontrollable level of accessing and using Facebook, which negatively affects other face-to-face social activities, studies, jobs, interpersonal relationships, and physical health [6,7].

Facebook addiction disorder is categorized by psychological factors such as salience, tolerance, mood modification, relapse, withdrawal, and conflict [8]. Salience is related to the mental state of continuously thinking about Facebook, whereas tolerance is related to the tolerance level of Facebook usage (eg, increase the time spent on Facebook to reach to the same effect that was initially experienced using Facebook). Mood modification is associated with whether Facebook affects current moods of the user, and relapse is linked with failed attempts of Facebook usage reduction. Meanwhile, withdrawal and conflict are related to negative conditions and effects because of failure in accessing Facebook, in which withdrawal is associated with negative conditions such as becoming restless because of failure in accessing Facebook, whereas conflict is linked with negative effects such as Facebook causing negative impacts on individuals’ current academic or professional life [2,9].

Previous research has revealed that Facebook addiction have caused negative psychological effects such as emotional problems, relational problems, health-related problems, and performance problems [9]. In terms of emotional problems, Facebook addiction has been revealed to cause negative mood alterations such as depression and anxiety [10], development of deficient self-regulation [11], as well as task avoidance and procrastination [12]. With regard to relational problems, Facebook addicts have experienced negative relationships in terms of family conflicts, impaired concentration at work or school, and problematic peer relationships, thus contributing to interpersonal relationship detriment [9,13]. With regard to health-related problems, Facebook addiction has also been associated with sleep difficulties such as insomnia and somatic problems as well as poorer sleep quality [9,14]. Meanwhile, for performance problems, addiction to Facebook has caused job losses and negative effects of self-reported work performance [9,14].

Facebook addiction has also been reported to affect higher education studies. In Turkey, a study on the effect of Facebook addiction on gender was carried out with 1257 Turkish university students [15]. The study’s findings revealed that male students had higher SNS addiction levels as compared with female students. In Poland, Facebook addiction was studied among 1157 students [6]. They discovered that Facebook addiction among Polish students was related to higher extraversion, narcissism, loneliness, and social anxiety and lower general self-efficacy. They also discovered that Facebook addiction was further related to impoverished well-being that included impaired general health, decreased sleep quality, and higher perceived stress. In the United States, an investigation was conducted with 274 university students in a statistics course, in which they examined the time distortion of social media addiction in at-risk students by intervention strategies such as prevention of Facebook use and self-control strategies [16]. They discovered that the at-risk group showed a significant upward time estimate bias when positively correlated with Facebook addiction scores. In Malaysia, Facebook addiction motives were studied with 380 postgraduates and undergraduates and the study revealed that motives that contribute to addiction are factors such as social interaction, passing time, entertainment, companionship, and communication. In another study, 441 Malaysian students were assessed on their addiction to Facebook [11]. They found out that factors such as religion, level of income, ego strength, and locus of control do not show significant influence on the risk of Facebook addiction, whereas time spent on Facebook contributed to higher addiction levels [17]. Nevertheless, these studies only mostly focused on motives of Facebook addiction level, and this shows that there is a lack of studies on the Web-based intervention systems for Facebook addiction.

### Objectives

Therefore, the main aim of this study was to investigate features of the Web-based intervention systems on management of Facebook addiction in postgraduate education. Considering the lack of knowledge on the development of the Web-based intervention features for Facebook addiction, the study was exploratory in nature [2,18]. The second aim was to investigate which intervention feature was most and least important for the management of addiction to Facebook. This could contribute to a better understanding of addiction prevention and therapeutic interventions of Facebook addiction. In the study, Web-based intervention features focused on features such as manual monitoring, manual limit, automatic notification, automatic limit, and automatic reward for learners to manage Facebook addiction. These features can be linked to an upcoming tool that is going to be introduced by Facebook called “Your Time on Facebook,” which allows for management of time on Facebook and includes an activity dashboard, a daily reminder, and management of notifications [19].

## Methods

### Participants

The participants of the survey were 200 postgraduates from a postgraduate support Facebook group called Doctorate Support Group (DSG). DSG is a support group that aims to provide a platform in supporting postgraduates to exchange ideas, expertise, and experiences in pursing their postgraduate education. The community has over 14,700 users who consist of postgraduates carrying out their studies and ex-postgraduate students who have completed their studies. The Facebook group applied a *community of practice* approach, in which the Facebook group provides a *shared knowledge* bank developed by the community members who have been associated with the community for a longer period. As newcomers join the group, the newcomers would learn from the *old-timers* who serve as *coaches* or *mentors* for the community. By participating in new activities and contributing to the community, the newcomers develop a new mastery of understanding and thus become recognized members (and later coaches) to give back to the community [3,20].

### Data Collection and Analysis Approaches

The data were collected via administration of online surveys on Facebook group of the DSG. The online survey included mock interfaces (ie, high-fidelity prototypes) of the intervention features. This includes Facebook features as reported by Facebook in their upcoming tool called “Your Time on Facebook” [19]. These upcoming features allow for Facebook users to manage their time on Facebook, which includes an activity dashboard, a daily reminder, and management of notifications. As such, in investigating the possible Web-based intervention features for Facebook addiction during postgraduate studies, mobile phone addiction intervention features by Lee et al were adapted, which included manual monitoring, manual limit, automatic notification, automatic limit, and automatic reward features as shown in Table 1 [21]. In prevention of excessive Facebook usage, 2 features were assessed, which were manual monitoring and manual control. The manual monitoring feature investigated whether postgraduates perceived features such as manually monitoring their usage patterns (including usage time, frequency, locations, and mood) could help in Facebook addiction management. Meanwhile, the manual limit investigated whether features such as manual limiting Facebook usage based on time, location, Facebook features, and moods could potentially assist them in intervention of Facebook addiction. With regard to automatic notification, the Web-based features touched on interventions with regard to automatic notification of excessive Facebook usage based on location, features, time, and mood. The automatic limit feature is linked to features that automatically limit the usage based on time, location, frequency of use, and mood. The final feature (automatic reward), which is related to Web-based intervention features, is related to rewarding mechanisms based on usage duration and frequency, location, and mood.

The study also investigated aspects of Facebook addiction during postgraduates’ studies using the Bergen Facebook Addiction Scale, which categorizes Facebook addiction disorder by psychological factors such as salience, tolerance, mood modification, relapse, withdrawal, and conflict [8]. As discussed before, salience is related to the mental state of continuously thinking about Facebook, whereas tolerance is related to tolerance level of Facebook usage. Mood modification is associated with whether Facebook affects current moods of the user, and relapse is linked with failed attempts of Facebook usage reduction, whereas withdrawal and conflict are related to negative conditions and effects because of failure in accessing Facebook [2,9]. Basic demographical data (ie, gender, age, and device usage on Facebook; current experience using Facebook; and Facebook usage frequency) were also assessed in the study.

The questionnaire developed based on Bergen Facebook Addiction Scale and Web-based intervention features (manual monitoring, manual limit, automatic notification, automatic limit, and automatic reward) was run through a content validation procedure to increase its level of validity. It was validated by 2 social networking analysis experts, 2 information technology experts, and a language lecturer. The content validation involved validation on aspects such as subject matter, technology, language, and measurement. This was conducted to verify that the questions for each variable were clear and concise. As a result, the online survey consisted of 48 items as measurements. The ethics committee of the Universiti Kebangsaan Malaysia approved the implementation of this study. We followed all national regulations and laws regarding human subjects’ research and obtained the required permission to conduct this study. Participants provided Web-based informed consent to participate.

**Figure 1:**
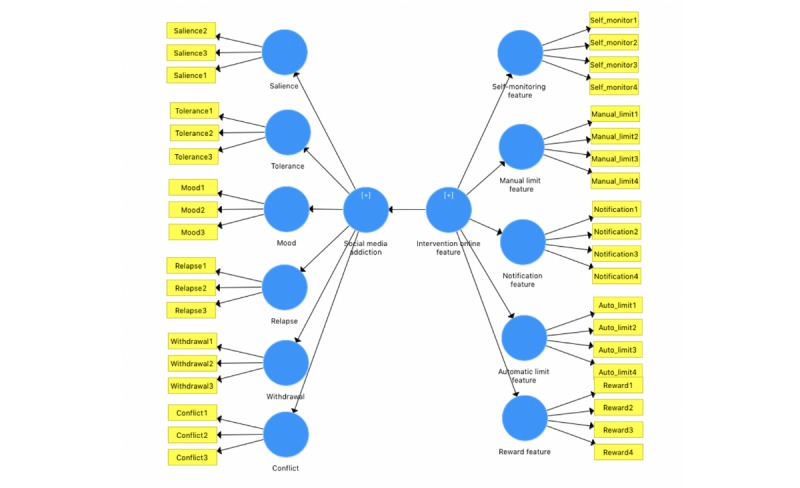
Model used for partial least square-structural equation modeling analysis for investigating the Web-based intervention features of Facebook addiction disorder.

The data collected were analyzed using partial least square-structural equation modeling (PLS-SEM). This allowed for exploratory investigation of the relationship between Web-based intervention features for Facebook addiction and aspects of Facebook addiction [22]. As the study implements exploratory research in nature, PLS-SEM was chosen. The technique allows for conducting predictions and explanation of target constructs rather than confirmatory analysis with the capability of small samples sizes and complex models. PLS-SEM analysis does not make any assumptions about underlying data; thus, 200 Web-based participants are enough [21,22]. Hence, the study focuses only on describing the structural model analysis results (via PLS-SEM model diagrams) with regard to loadings of each construct and does not report on measurement model analysis results. The results of loadings would help in understanding the most and least important intervention features for Facebook addiction and the strongest and weakest causes of Facebook addiction among postgraduates. The software used to run PLS-SEM analysis is SmartPLS version 3.2.7 by SmartPLS GmbH. The model used in the analysis is illustrated in Figure 1.

**Table 1:** The constructs and respective indicators of the Web-based intervention features for Facebook addiction disorder.

Construct	Indicator
Self-monitoring feature (IF^a^_Self-monitoring)	Track Facebook usage time Track Facebook frequently used features Track location of Facebook usage Track mood while using Facebook
Manual limit feature (IF_Manual limit)	Manually limit Facebook usage based on time Manually limit Facebook usage based on location Manually limit Facebook usage based on features Manually limit Facebook usage based on mood
Notification feature (IF_Notifcation)	Notification of excessive Facebook usage based on time Notification of excessive Facebook usage based on location Notification of excessive Facebook usage based on features Notification of excessive Facebook usage based on mood
Automatic limit (IF_Auto-limit)	Automatically limit Facebook usage based on time Automatically limit Facebook usage based on location Automatically limit Facebook features based on frequency of use Automatically limit Facebook usage based on my mood
Reward feature (IF_Reward)	Provide reward based on Facebook usage time Provide reward based on Facebook usage location Provide reward based on Facebook feature frequency Provide reward based on mood using Facebook

^a^IF: intervention Web-based feature.

## Results

### Demographical Findings

Most of the 200 participants of the online survey were female (77.5%, 155/200), whereas the remaining 22.5%, 45/200 were male. The age range was quite diverse as it included individuals aged between 23 years and 51 years. For devices used to access the Facebook, 9%, 18/200 of them used only computers, 36.5%, 73/200 used only mobile phones, and 53.5%, 107/200 of them used both computers and mobile phones. The survey also gained data regarding current experience of Facebook usage. The data revealed that 86.5%, 173/200 of them have used Facebook for more than 4 years, 11.5%, 23/200 of them have used Facebook for 3 to 4 years, whereas 2.0%, 4/200 of them have used Facebook for 1 to 2 years. In addition, Facebook usage frequency was also obtained. Findings indicated that most of them (83.0%, 166/200) access Facebook every day, whereas the others access it either 2 to 3 times a week or 4 to 5 times a week. Findings also revealed that 21.5%, 43/200 of them access Facebook more than 10 times a day, 20.0%, 40/200 access it 7 to 10 times a day, 24.0%, 48/200 access it 4 to 7 times a day, and 34.5%, 69/200 of them access it once a day.

### Results on Partial Least Square-Structural Equational Modeling Results: Facebook Addiction

**Figure 2:**
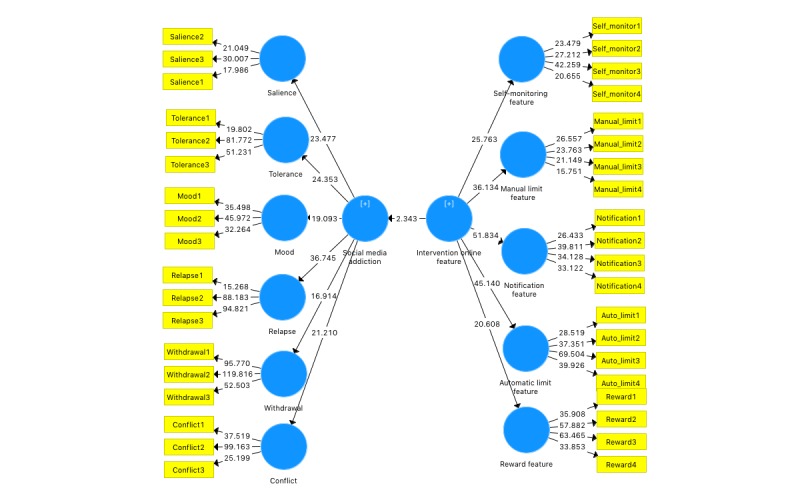
Bootstrapping results of the partial least square-structural equation modeling analysis.

The findings of the measurement model analysis showed that the indicators of the constructs achieved internal consistency reliability, convergent validity, and divergent validity. The results are summarized in Tables 2-4. The findings of the structural measurement model analysis revealed that there are 6 addiction factors that are related to Facebook addiction in postgraduate studies, as shown in Figure 2. They are mood modification, withdrawal, tolerance, salience, conflict, and relapse, which received loadings of 0.5 and above [22]. This indicates that all the indicators (eg, FB_Salience1) are related to their respective constructs (eg, FB_Salience). These results corroborate with the works of Griffiths, Kuss and Griffiths, and Andreassen, where the studies revealed that these 5 levels contribute to SNS addiction, in this case, Facebook addiction [9,22,23].

**Table 2:** Internal consistency reliability results.

Indicator	Average variance extracted (AVE)	Composite reliability (CR)	*R* ^2^	Cronbach alpha
FB^a^_Conflict	0.732	0.891	0.593	.815
FB_Mood modification	0.722	0.886	0.394	.808
FB_Relapse	0.725	0.886	0.666	.804
FB_Salience	0.590	0.812	0.559	.659
FB_Tolerance	0.739	0.894	0.542	.821
FB_Withdrawal	0.845	0.942	0.511	.908
IF^b^_Auto-control	0.740	0.919	0.721	.882
IF_Manual Control	0.583	0.847	0.664	.759
IF_Notification	0.692	0.900	0.797	.852
IF_Reward	0.786	0.936	0.668	.909
IF_Self-monitoring	0.630	0.872	0.599	.803

^a^FB: Facebook addiction factor.

^b^IF: intervention Web-based feature.

**Table 3:** Convergent validity results.

Construct and indicator		Loading	Average variance extracted (AVE)	Composite reliability (CR)
**FB^a^_Conflict**			**0.732**	**0.891**
	FB_Conflict1	0.845		
	FB_Conflict2	0.923		
	FB_Conflict3	0.794		
**FB_Mood modification**			**0.722**	**0.886**
	FB_MoodModification1	0.837		
	FB_MoodModification2	0.865		
	FB_MoodModification3	0.847		
**FB_Relapse**			**0.725**	**0.886**
	FB_Relapse1	0.709		
	FB_Relapse2	0.910		
	FB_Relapse3	0.918		
**FB_Salience**			**0.590**	**0.812**
	FB_Salience1	0.705		
	FB_Salience2	0.804		
	FB_Salience3	0.792		
**FB_Tolerance**			**0.739**	**0.894**
	FB_Tolerance1	0.765		
	FB_Tolerance2	0.922		
	FB_Tolerance3	0.884		
**FB_Withdrawal**			**0.845**	**0.942**
	FB_Withdrawal1	0.928		
	FB_Withdrawal2	0.948		
	FB_Withdrawal3	0.880		
**IF^b^_Auto-control**			**0.740**	**0.919**
	IF_AutoControl1	0.807		
	IF_AutoControl2	0.876		
	IF_AutoControl3	0.909		
	IF_AutoControl4	0.845		
**IF_Manual Control**			**0.583**	**0.847**
	IF_ManualControl1	0.815		
	IF_ManualControl2	0.790		
	IF_ManualControl3	0.769		
	IF_ManualControl4	0.670		
**IF_Notification**			**0.692**	**0.900**
	IF_Notification1	0.809		
	IF_Notification2	0.858		
	IF_Notification3	0.826		
	IF_Notification4	0.834		
**IF_Reward**			**0.786**	**0.936**
	IF_Reward1	0.856		
	IF_Reward2	0.901		
	IF_Reward3	0.918		
	IF_Reward4	0.869		
**IF_Self-monitor**			**0.630**	**0.872**
	IF_SelfMonitor1	0.768		
	IF_SelfMonitor2	0.821		
	IF_SelfMonitor3	0.840		
	IF_SelfMonitor4	0.741		

^a^FB: Facebook addiction factor.

^b^IF: intervention Web-based feature.

**Table 4:** Divergent validity results (Fornell-Larcker Criterion).

Construct	FB^a^_Conflict	FB_Mood modification	FB_Relapse	FB_Salience	FB_Tolerance	FB_Withdrawal	IF^b^_Auto-control	IF_Manual Control	IF_Notifi-cation	IF_Reward	IF_Self-monitoring
FB_Conflict	0.856	—^c^	—	—	—	—	—	—	—	—	—
FB_Mood modification	0.320	0.850	—	—	—	—	—	—	—	—	—
FB_Relapse	0.684	0.359	0.851	—	—	—	—	—	—	—	—
FB_Salience	0.483	0.406	0.505	0.768	—	—	—	—	—	—	—
FB_Tolerance	0.429	0.452	0.485	0.529	0.860	—	—	—	—	—	—
FB_Withdrawal	0.428	0.344	0.497	0.464	0.371	0.919	—	—	—	—	—
IF_Auto-control	0.160	0.251	0.226	0.106	0.209	0.038	0.860	—	—	—	—
IF_Manual Control	0.157	0.153	0.217	0.079	0.177	–0.015	0.626	0.763	—	—	—
IF_Notification	0.224	0.270	0.229	0.080	0.141	–0.005	0.718	0.697	0.832	—	—
IF_Reward	0.200	0.241	0.174	0.143	0.154	0.002	0.599	0.521	0.668	0.886	—
IF_Self-monitoring	0.115	0.162	0.166	0.141	0.140	0.012	0.539	0.610	0.597	0.545	0.794

^a^FB: Facebook addiction factor.

^b^IF: intervention Web-based feature.

^c^Not applicable.

The results also indicated that the Facebook addiction construct with the highest loading was relapse (0.666), followed by conflict (0.593), salience (0.559), tolerance (0.542), and withdrawal (0.511), as in Table 5. The lowest loading was obtained by the mood modification construct that was 0.394. The results signify that the strongest Facebook addiction factors in postgraduates’ studies are relapse and conflict, whereas the 2 weakest levels are mood modification and withdrawal. This contradicts the results of the studies by Koc and Gulyagci as well as Balakrishnan and Shamim, where the former authors revealed that mood modification and conflict are the most frequent symptoms of Facebook addictive usage among university students, whereas the latter authors revealed that salience, loss of control, and withdrawal are the main indicators of Facebook addiction among students [24,25].

**Table 5:** Structural model results.

Hypothesis	*R* ^2^	Beta	SE	*t* value	Decision
FB^a^_Conflict ≥ FB Addiction	0.593	.238	0.020	12.144^b^	Support
FB_Mood modification ≥ FB Addiction	0.394	.190	0.022	8.680^b^	Support
FB_Relapse ≥ FB Addiction	0.666	.251	0.018	13.929^b^	Support
FB_Salience ≥ FB Addiction	0.559	.184	0.017	10.672^b^	Support
FB_Tolerance ≥ FB Addiction	0.542	.229	0.019	12.384^b^	Support
FB_Withdrawal ≥ FB Addiction	0.511	.256	0.020	12.966^b^	Support
Intervention Features ≥ IF^c^_Auto-control	0.721	.851	0.024	35.550^b^	Support
Intervention Features ≥ IF_Manual Control	0.664	.816	0.036	22.926^b^	Support
Intervention Features ≥ IF_Notification	0.797	.891	0.025	36.247^b^	Support
Intervention Features ≥ IF_Reward	0.668	.820	0.037	22.182^b^	Support
Intervention Features ≥ IF_Self-monitoring	0.599	.776	0.045	17.367^b^	Support

^a^FB: Facebook addiction factor.

^b^*P*<.05.

^c^IF: intervention Web-based feature.

These results could be caused by the fact that Facebook addiction factors could potentially be explained by a process in which a Facebook addict goes through levels of addictions that ends with relapse and conflict levels, where they attempt to reduce Facebook time but fail to do so (relapse) and ignore their studies and people (conflict) [9]. This can also be related to Facebook usage frequency of postgraduates in this study, where most of them (83%) accessed Facebook every day and 65.5% of them accessed Facebook more than 4 times a day. In addition, students who are Facebook addicts have possibly never deactivated their accounts before showing their high Facebook addiction level [25]. Furthermore, Cabral reported that the majority of participants in their study reported failed attempts of social media usage reduction [26].

The findings also revealed that 2 of the relapse construct’s indicators FB_Relapse2 and FB_Relapse2 obtained the highest loadings. The indicators were related to relapse in decision making and actions on Facebook usage, which included “decided to use Facebook during your postgraduate studies less frequently, but not managed to do so” and “tried to cut down on the use of Facebook during your postgraduate studies without success.” This is in line with the findings of Brailovskaia and Margraf’s study that investigated Facebook addiction disorder among German students [2]. They discovered that Facebook addiction factors fully mediated the association between narcissism and stress systems, and the highest positive association was with 3 factors, which were relapse, withdrawal, and salience. From that study, they revealed that users who are narcissist tend to spend more time thinking about Facebook because of Web-based self-presentation, interaction, and reflections in the social networking platform, thus causing them to be vulnerable to Facebook addiction and be in a state of relapse.

## Discussion

### Discussion on Partial Least Square-Structural Equational Modeling Results: Web-Based Intervention Features

The findings of the structural measurement model analysis show that 6 Web-based intervention features are related to Web-based intervention and Facebook addiction in postgraduate studies. The factors are manual monitoring feature, manual limit feature, automatic notification feature, automatic limit feature, and automatic reward feature, which obtained loadings above the 0.5 cut-off point for loadings, as shown in Tables 2-4 [22]. This indicates that all the indicators (eg, IF_manual_monitoring1) are related to their respective constructs (eg, manual monitoring).

The results also revealed that the Web-based intervention feature for postgraduate education that received the highest loading was automatic notification feature (0.797), followed by automatic limitation feature (0.721), automatic reward feature (0.668), and manual limitation feature (0.664). The lowest loading gained was by manual monitoring feature (0.599), as shown in Table 6. The results suggest that the 5 intervention features could be used in management or intervention of Facebook addiction in postgraduate education. In other words, this indicates that postgraduates prefer to be notified of their Facebook usage (notification) and then be automatically managed or restricted to Facebook based on time, frequency, and location of Facebook usage as well as mood during Facebook access. Although this may seem like a straightforward solution in managing Facebook addiction, it may not be the case. This can be related to a study on Facebook addiction with regard to active Facebook use (ie, using Facebook for communication) and passive Facebook use (ie, using Facebook to consume content) [5]. They discovered that passive Facebook use was related to daily life events. Interestingly, the study revealed that participants of the study increased Facebook usage following positive life events instead of negative ones. In other words, passive Facebook use is less likely to be associated with escapism as users have decreased level of passive Facebook use when faced with problems as compared with positive experiences.

This can further be related to another relevant study, where the study indicated that Facebook addiction is related to narcissism and stress systems [2]. Linking the 2 studies together, this indicates that postgraduates who have Facebook addicts are more likely to use Facebook to consume information-related positive life events, in this case related to academic success, rather than using Facebook for escapism related to negative emotions. On that note, it would be interesting for future Web-based intervention features to include the option for passive and active Facebook use and relate it with positive and negative life events in postgraduate education. In terms of the automatic reward feature, this suggests that rewards (eg, rewards systems in gaming, such as scores, or virtual currencies—refer to Yen’s study [27]) could be used as an intervention measure for addicts. Although results revealed that manual control and self-monitoring were the least important intervention features, both are still essential as they allow postgraduates to monitor their Facebook usage levels and manually control/manage Facebook features based on time, location, and feature usage as well as inputting their moods.

**Table 6:** Coefficient of determination (*R*^2^) test.

Hypothesis	*R* ^2^
FB^a^_Relapse ≥ FB Addiction	0.666
FB_Conflict ≥ FB Addiction	0.593
FB_Salience ≥ FB Addiction	0.559
FB_Tolerance ≥ FB Addiction	0.542
FB_Withdrawal ≥ FB Addiction	0.511
FB_Mood modification ≥ FB Addiction	0.394
Intervention Features ≥ IF^b^_Notification	0.797
Intervention Features ≥ IF_Auto-control	0.721
Intervention Features ≥ IF_Reward	0.668
Intervention Features ≥ IF_Manual Control	0.664
Intervention Features ≥ IF_Self-monitoring	0.599

^a^FB: Facebook addiction factor.

^b^IF: intervention Web-based feature.

### Conclusions, Implications, and Future Directions

The study discovered 6 Facebook addiction factors (relapse, conflict, salience, tolerance, withdrawal, and mood modification) and 5 intervention features (notification, auto-control, reward, manual control, and self-monitoring) that could be used in management of Facebook addiction in postgraduate education. The study also revealed that relapse is the most important factor and mood modification is the least important factor. Furthermore, findings indicated that notification was the most important intervention feature, whereas self-monitoring was the least important feature. This study’s findings, with regards to social media addiction factors and Web-based intervention features, could assist future developed and educators in the development of Web-based intervention tools for Facebook addiction management in postgraduate education. In addition, PLS-SEM was used as a statistical approach to verify the relationship between social media addiction disorder management and Web-based intervention features, which contributes to the field in terms of the higher education field, particularly in postgraduate education.

Future directions in this area are as follows. First, the addiction factors and intervention features were only tested in postgraduate educational settings. It would be interesting to investigate whether the findings corroborate or contradict with these findings in other educational settings, which include undergraduate, primary, and secondary education as well as long-life learning settings [28]. Second, most of the respondents were studying in local higher education institutions. It would be worth replicating the study with a larger sample with a more diverse span of international higher educational institutions [29,30]. Finally, it would be interesting to combine the results with social network analysis as to indicate whether social network patterns (in egocentric diagrams) could be used in the management of Facebook addiction [3] as well as other Web-based approaches [31-36].

## Abbreviations

DSG: Doctorate Support Group
FB: Facebook addiction factor
IF: intervention Web-based feature
PLS-SEM: partial least square-structural equation modeling
SNSs: social networking sites
